# Marbled murrelet habitat suitability in redwood timberlands of Northern Coastal California utilizing LiDAR-derived individual tree metrics

**DOI:** 10.1371/journal.pone.0307726

**Published:** 2025-02-20

**Authors:** David W. Lamphear, Keith A. Hamm, Desiree A. Early, Trent L. McDonald

**Affiliations:** 1 Green Diamond Resource Company, Korbel, CA, United States of America; 2 McDonald Data Sciences LLC, Laramie, WY, United States of America; National Museums of Kenya, KENYA

## Abstract

The marbled murrelet (*Brachyramphus marmoratus*) is a threatened seabird found from Southern California to Alaska that forages at sea but nests in near coastal forests. Marbled murrelet nesting habitat is generally comprised of old-growth or mature forests with large trees having platforms suitable for nesting. Here, we estimate a habitat suitability model (HSM) that relates evidence of nesting to characteristics of putative trees derived from high resolution light imaging detection and ranging (LiDAR) data. Our study area in Northern California contained stands of old-growth forests on state, federal, and private lands but was predominated by private second-growth redwood and Douglas-fir timberlands. We estimated a two-sample HSM using Maxent software and implemented objective and repeatable covariate selection, model evaluation, and classification methods. Our HSM predicts relative likelihood of occupancy using predicted Habitat Suitability Index (HSI) values that we then classify into five habitat classes based on a novel use of the predicted to expected (P/E) ratio curve. From HSI predictions, we identified patches of murrelet habitat and estimated concave polygons surrounding individual trees within and proximately close to each patch. These methods provide repeatable boundaries for identification of patches and important individual trees based on HSI. Patches with greater relative probability of occupancy were characterized by high densities of 60-meter and taller trees, a large sum of heights for 50-meter and taller trees, and high values of standard deviation of 50-meter and taller trees. During hold-out model evaluations, our HSM showed extremely high fidelity for known patches with indirect evidence of nesting based on occupancy.

## Introduction

The United States Fish and Wildlife Service listed the marbled murrelet (*Brachyramphus marmoratus*) as threatened under the federal Endangered Species Act in 1992. That same year, the state of California listed the bird as endangered under the California Endangered Species Act. At the time, scientists knew that this seabird foraged offshore and nested in nearshore older forests along the Pacific Coast of the United States and Canada. Since then, several studies have investigated landscape and stand-level characteristics that comprise suitable marbled murrelet nesting habitat. Several studies of landscape scale habitat use found associations between marbled murrelet nesting and large amounts of unfragmented old-growth or mature forests [1–3]. Other studies characterized marbled murrelet nesting habitat as un-fragmented old-growth low elevation sites within the fog zone near other marbled murrelet nesting sites [4–7]. Still other studies found a negative association between marbled murrelet nest sites and increasing amounts of forest fragmentation, edge, and areas of logged and immature forests [1, 6, 8–12].

At a smaller scale, individual forest stands containing marbled murrelet nests tend to have high densities of large trees (minimum diameter at breast height of 120 centimeter (4 feet)) with multiple platforms, multiple canopy layers, canopy gaps, high epiphyte thickness, high epiphyte percentages, and greater tree heights [5, 12–17]. In one Oregon study, observed nest trees contained 8-92 platforms [3]. Other Oregon studies documented few nests in non-old growth trees (60-80 years) with dwarf mistletoe, deformations, or structural elements that support a nest [17, 18]. In addition, canopy complexity is an important component of nesting stands because openings are required for landing and takeoff [9, 11, 19]. In California, marbled murrelets nest primarily in old growth redwood and Douglas-fir forests [4, 12, 16, 20–22]. Carter and Erickson [23] documented marbled murrelets nesting up to 40 kilometers inland from the northern California coast. This literature provides a basis for identifying potential marbled murrelet habitat using stand-level criteria, but we wanted to develop a reliable tool for identifying a continuum of probable marbled murrelet habitat across a landscape at scales smaller than the typical stand.

In our study, we had three objectives. First, as part of a federal habitat conservation plan (HCP), we developed a correlative habitat suitability model (HSM) for marbled murrelets to identify, at the patch and study area scale, forested areas that correlate well with areas where marbled murrelets have exhibited indirect evidence of nesting (“occupied behaviors”; [24]) or where other observations provided direct evidence of nesting (e.g., radio telemetry or tree climbing). Second, we developed habitat classes based on the predicted to expected (P/E) ratio curve of HSI values [25, 26]. Third, we estimated concave polygons surrounding the patch and individual trees that influence nesting potential of an area and used these polygons to delineate management-friendly patch boundaries and associated habitat quality predictions. These patch boundaries would include trees in the core area of nesting patches but also included peripheral large trees that contributed to nesting suitability. To our knowledge, this would be the first successful use of LiDAR derived height and density of individual trees to model marbled murrelet nesting habitat.

## Study area

**Fig 1:**
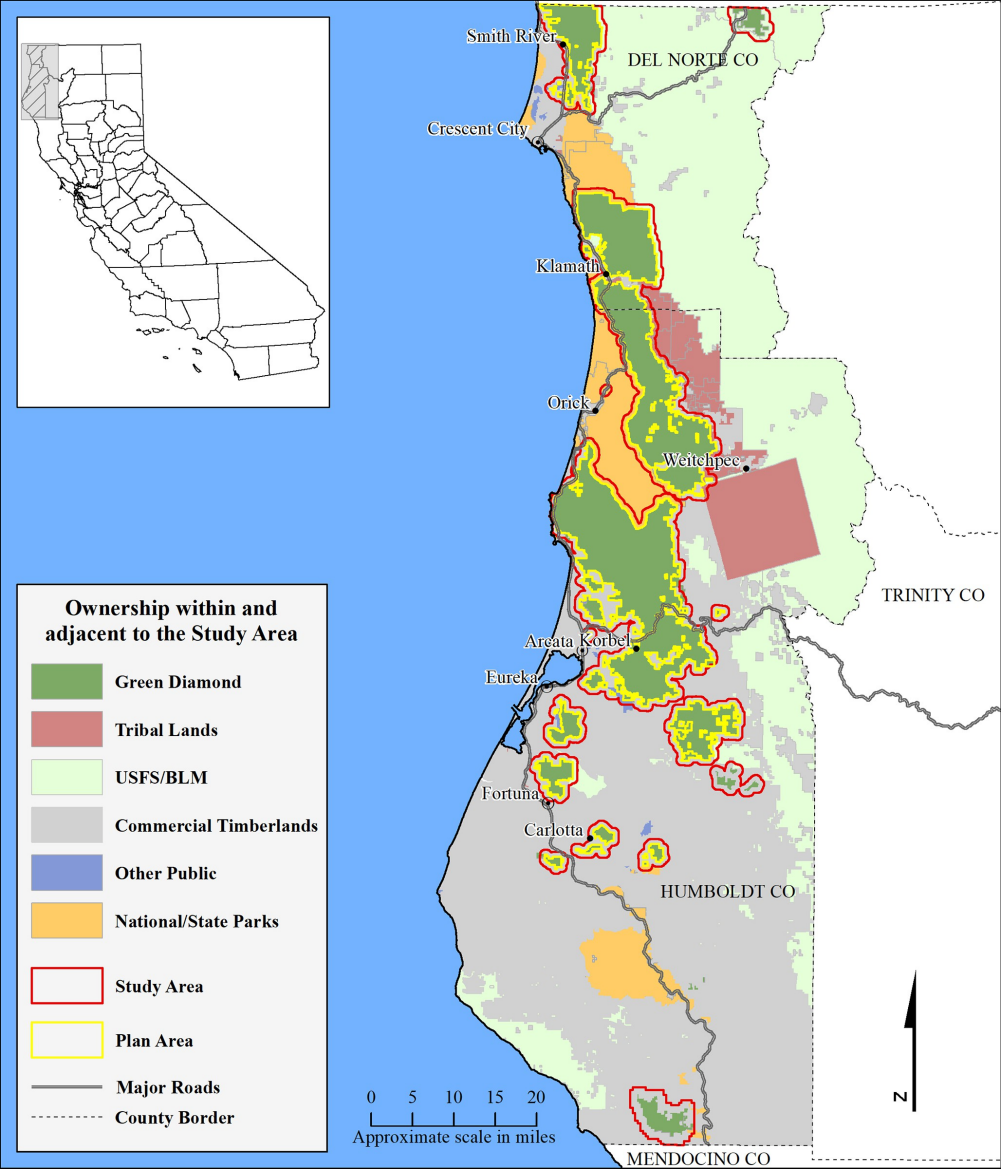
Ownership within the study area. The study area, composed of Green Diamond timberlands in Humboldt and Del Norte Counties, and a surrounding 0.5-mile buffer that contained State, Federal, Tribal, other Private Commercial timberlands, and Private non-timberlands. The Plan Area comprised a subset of Green Diamond Timberlands.

Our study area, comprised of Green Diamond Resource Company lands plus a ½ mile buffer, was located on a mix of commercial timberlands, state, federal, and Tribal lands, with a negligible proportion of public or private non-timberlands, in Humboldt and Del Norte Counties, California (Fig 1). The study area was limited to the extent of our high-resolution LiDAR data collected in 2017/2018. Timberland ownership within the 238,355-hectare (588,987-acre) study area was composed of Green Diamond Resource Company (63.8%), other Private (25.0%), State or Federal including Redwood National and State Park, U. S. Forest Service and Bureau of Land Management (8.6%), and Tribal including Yurok and Hoopa lands (1.8%). The remainder (< 1%) were public or private non-timberlands. Hereafter, we will use the term ”private timberlands” for both Green Diamond and non-Green Diamond timberlands within the study area. The portion of our study area included in Green Diamond’s HCP application is 145,100 hectares (358,549 acres). Hereafter, we refer to this portion of the study area as the “Plan Area”.

The entire study area was within 50 kilometers (31.1 miles) of the Pacific Ocean, and elevation ranged from five to 1550 meters (5,085 ft). The present-day climate on the North Coast of California is characterized as temperate Mediterranean. Along the Pacific Ocean, winters are generally mild and rainy, and summers are mild, cool, and dry. Further inland, the weather varies from the oceanic climate and temperatures increase throughout the year into a hot summer. Variation in air temperature is generally low along the coast as maritime influence dominates, with decreasing affect inland. Yearly minimum air temperature varies spatially across the study area from -1.6°Celsius (29.5°Fahrenheit) to 5.3° Celsius (41.6° Fahrenheit) with cooler minimum temperatures concentrated inland and in the north. Yearly maximum air temperature varies similarly from 17.5° Celsius (63.6° Fahrenheit) to 35° Celsius (95.0° Fahrenheit) with warmer temperatures concentrated inland and in the broad river valleys nearer the coastline. Annual precipitation occurs mostly as rain from November through April averaging 198 centimeters (78 inches) and varies from 106 centimeters (42 inches) near the coast to 320 centimeters (155 inches) in the coastal mountains [27]. Substantial and persistent snow occurs at elevations above 1000 meters (3,280 feet).

In our study area, coast redwood (*Sequoia sempervirens*) forest dominated in the coastal areas and at lower elevations. Douglas-fir (*Pseudotsuga menziesii*) replaced redwood as elevation and distance from the coast increased. Hardwoods exist as pure stands or common forest components. Red alder (*Alnus rubra*) and maple (*Acer macrophyllum*) dominated in coastal mesic sites, while tanoak, madrone (*Arbutus menziesii*) and giant chinquapin (*Chrysolepis chrysophylla*) occurred at higher, more xeric sites.

Private timberlands in the study area have been subjected to timber harvest for more than a century. Forests on private timberlands consist primarily of second and third-growth trees ranging in age from recently harvested to approximately 120 years. Private timberlands contain small and scattered patches of unharvested or partially harvested old growth forest (< 0.2% of private timberlands). Larger intact old growth forests occur on state and federal lands. State and federal lands in the study area consist of some of the largest old growth coastal redwood forests remaining within the California range of the marbled murrelet [28].

## Methods

We estimated the two-sample HSM [29, 30] for murrelet nesting habitat using Maxent software [31]. Our two-sample HSM compared environmental conditions (i.e., habitat characteristics) at a sample of “used” locations to those at a sample of “available” locations. We estimated our HSM using the two-sample used-available methodology [29], also referred to as presence-background [31, 32], because we had extensive GIS coverage of known occupied patches but no widespread objective surveys of putative nest trees to establish use and non-use. We selected covariates to include in the HSM using an objective and repeatable four-step procedure. We validated the model by inspecting the magnitude and variation of performance measures over bootstrap replicates drawn from the raw data. We derived habitat classes by objectively identifying changes (“kinks”) in the predicted-expected ratios [26] over an increasing range of suitability. We estimated concave polygons surrounding the patch and individual trees that influence nesting potential of an area and used these polygons to delineate practical and identifiable stand boundaries and associated habitat quality predictions. These practical and identifiable patch boundaries included trees in the core area of nesting stands but also included peripheral large trees that contributed to nesting suitability.

### Used habitat polygons

We constructed polygons of “use” as forest areas with indirect evidence of nesting and labeled them “occupied habitat polygons”. We constructed “used” polygons, rather than used trees or points, due to the paucity of data on known nest locations within our study area. Prior studies faced a similar paucity of nest locations and, like us, defined “occupied behaviors” in a stand as an indication of potential nesting in the stand [24, 33, 34]. We reviewed available survey data collected from Federal and State parks, Bureau of Land Management reserves, private unharvested old growth forests, private partially harvested old growth, and private young (< 70 years old) forests with residual old growth trees containing “occupied behaviors” within the study area [16, 35, 36]. Surveys were conducted by various certified observers across multiple years over the past three decades for different purposes including monitoring efforts on public lands under the Northwest Forest Plan, Habitat Conservation Plans on private lands, and pre-project surveys on private timberlands. We included as “used” polygons all confirmed occupied patches. In total, forty-eight habitat patches, ranging from 0.1 to 463.4 hectares with a mean of 51.2 hectares were included and sampled as use areas.

Murrelet occupied behaviors in forest stands and contiguous habitat were established by three methods. Occupancy was established using audio-visual surveys conducted according to inland survey protocols for marbled murrelets that account for imperfect detection [24, 37], using radar surveys [38], and using direct evidence of murrelet nesting reported by other studies [21, 22]. The audio-visual inland survey protocol dictated multiple site visits (≥ 5) over a minimum of two years. During these surveys, occupied behaviors were noted such as stationary vocalizations from a tree, landing in a tree, flights below and through the forest canopy, and circling flights below or above the forest canopy. If murrelet occupied behaviors were observed, we assigned “used” status to the area. If surveys on private timberlands failed to observe occupied behaviors, the forested areas were cleared for harvest under California timber harvest plan permits [39]. The audio-visual surveys provided evidence of non-use when surveys failed to observe occupied behavior. Many of these non-use areas were subject to timber harvest prior to acquisition of the LiDAR data, and we could not derive accurate LiDAR covariates in these areas. We included these cleared for harvest areas among the “available” habitat.

When occupied behaviors were observed, the boundaries of the patch associated with the occupied behavior were established based on notable differences in forest age (or structure) such as between unharvested or lightly harvested old growth habitat (> 200 years old) and adjacent young managed forests generally less than 70 years old. We also considered the concept of contiguous habitat from surveyed areas [24]. On Green Diamond timberland, we included 24 patches that contained historic indirect evidence of nesting, and three patches with recent indirect evidence of nesting. We established the boundaries of the three recent patches using spatial kernel density estimation (95% KDE) of audio/visual detections. Forty-eight total used habitat patches, ranging from 0.1 to 463.4 hectares with a mean of 51.2 hectares were included and sampled from.

We utilized pre-existing data for these analyses such that field site access was not required. Data utilized was either publicly available (i.e., data from federal and state entities) or permission was obtained for use of data collected from private lands (i.e., Green Diamond Resource Company and Humboldt Redwood Company). Since we utilized pre-existing data, no permits were required for field site access or survey work.

## Balanced sampling – “Used” and “Available” points

**Fig 2:**
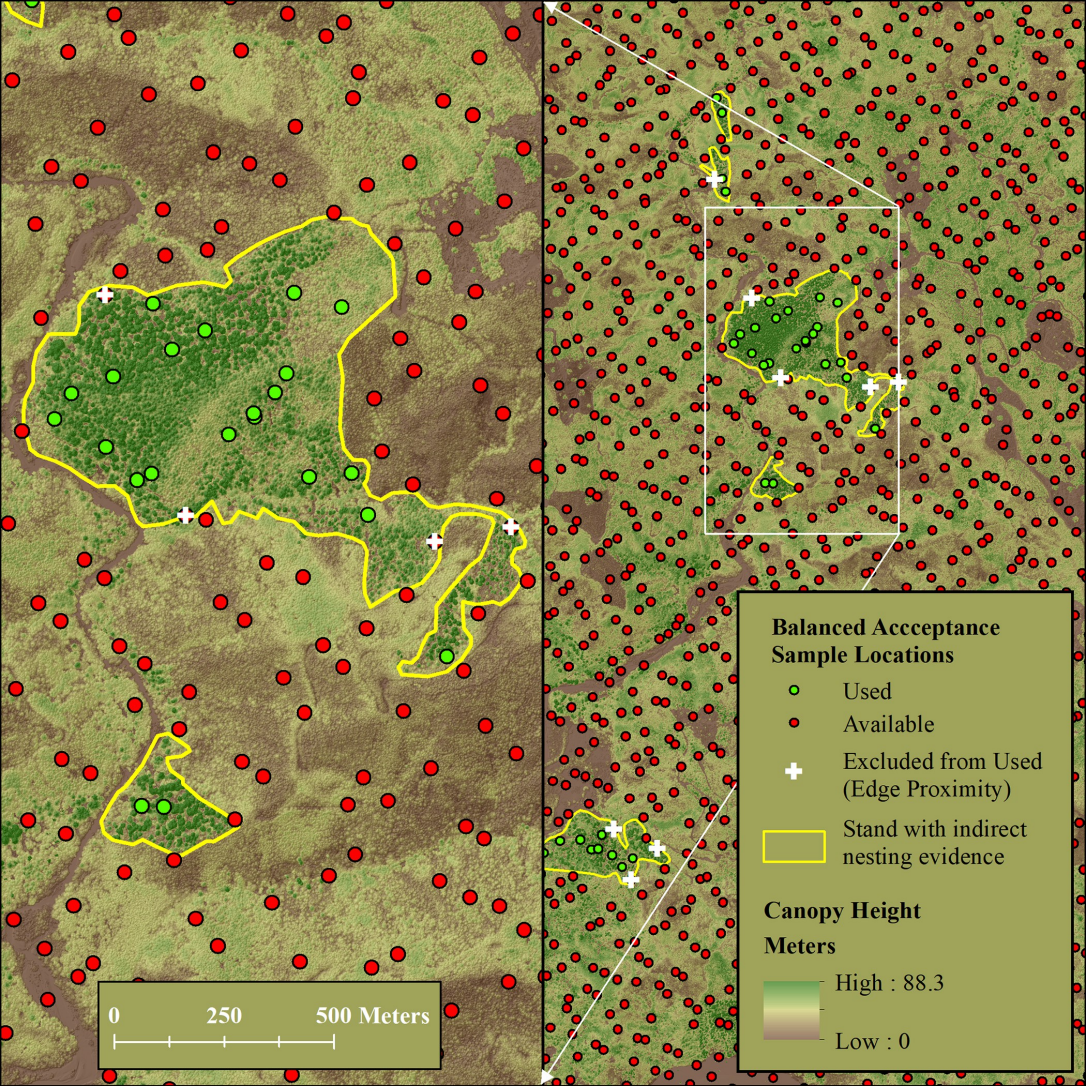
Balanced acceptance sampling (BAS). Illustration of Balanced Acceptance Sample (BAS) within the study area. We sampled “used” points (green dots) and “available” locations (both green and red dots) at an average density of one point per 5 acres. We excluded from the sample of “used” points any location within 20 meters inside the boundary of a patch with indirect evidence of nesting to reduce moving window edge effects (white crosses). We classified all BAS points (inside, outside, and near boundary) as “available”. Dark brown areas with low canopy height are prior clear cuts of varying age.

After establishing “used” polygons, we drew a single balanced acceptance sample (BAS) [40, 41] containing 117,804 locations across the entire 238,310-hectare (588,838-acre) study area. Our BAS had an average density of one sample point per 2.02 hectares (5-acres). We extracted all BAS points falling inside “used” polygons, but then excluded those within 20 meters of their patch boundary. We removed points inside but near the patch boundary to reduce edge effects (bias) in LiDAR-derived moving window covariates. We chose the inner exclusion zone distance of 20 meters, which is less than the 35.89-meter radius of our smallest 1-acre moving window, to maximize the number of “used” locations while balancing our concern over edge effect bias. We identified a total of 1,080 BAS points falling inside the “used” polygons after excluding those within 20 meters of patch boundaries (Fig 2). We considered these 1,080 points as “used”, while we considered the totality of BAS points (117,804 points) as “available”.

### LiDAR data

LiDAR data was collected over the 238,355-hectare study area (Fig 1) from December 2017 through February 2018 using a Cessna Caravan aircraft carrying a Riegl VQ-1560i LiDAR sensor. Wave form data was collected and discretized in real-time on board the aircraft yielding up to 14 returns per pulse. Survey altitude above ground (AGL), speed, swath overlap, and central wavelength of the laser were 1,306 meters, 120 knots, 55%, and 1064 nanometers, respectively. Specifications called for a minimum density of 30 pulses/m^2^. Our realized pulse density averaged greater than 40 pulse/m^2^, with a maximum of 560 returns per m^2^. Ninety-five percent of returns had horizontal and vertical accuracy of ≤ 20 centimeters. We have found that pulse densities greater than the typical United States Geological Survey standard (>=8/m^2) are necessary to resolve individual trees using local maxima methods. This is especially true where redwood stump sprouts produce tree leaders within 1 meter of each other. Raw LiDAR data consisted of the rectified and classified 3-dimensional location of every return collected by the sensor.

We processed the raw LiDAR data in LAStools [42] which among other things allowed us to discretize the (*x*, *y*, *z*) point cloud into “voxels” (a 3-dimensional pixel extending from ground to AGL), measure the distribution of points inside voxels (5-meter by 5-meter footprint), and develop a 0.25-meter resolution Digital Surface Model (DSM) for the canopy surface. Voxel metrics were calculated using LAStools and described below. We derived tree approximate objects (TAOs) by applying a local maxima process to the canopy DSM that ultimately extracted the (*x*, *y*) location and height (*z*) of the TAO [43–46].

### Covariates

We compiled eighty HSM covariates on a 5-meter grid covering the entire study area. We grouped these eighty covariates into six general themes [47] based on their hypothesized importance as predictors of murrelet nesting habitat (S1 Table in S1 File). The “Biographic/Geographic” group included 14 covariates such as elevation, distance to coast, aspect, and solar radiation. The “Climate” group included 6 covariates such as annual precipitation, air temperature, growing degree days, and whether a location was in the zone of coastal influence. The “LiDAR Canopy Metrics” group included 10 canopy metrics including the number of returns in each 5-meter by 5-meter voxel footprint, average return height above ground (a measure of central canopy height), and variation in returns within a given voxel. The “LiDAR Percentile” group included 19 variables measuring the height inside each voxel from which a certain percentage of the returns were received. We derived 16 TAO based statistics in the “TAO Point Statistics” group such as the minimum, mean, maximum, sum and standard deviation of TAOs ≥ 50 meters in height within 1- and 5-acre circular plots surrounding the (*x*, *y*) location of a voxel [48]. The “TAO Point Density” group included 15 covariates such as number of TAOs ≥ 50 meters within a 1- and 5-acre circular plot centered on the voxel’s (*x*, *y*) location. Only covariates in the “TAO Point Statistics” and “TAO Point Density” groups were averaged over spatial areas and subject to bias near patch edges, which we mitigated by eliminating used points within 20 meters of patch boundaries. Spatial reference for our 5-meter resolution covariate grids was NAD83, UTM Zone 10.

### Model selection

We investigated pairwise correlations among covariates by computing Pearson’s R^2^, Cramer’s V, and box plots. From these, we identified pairs and groups of correlated variables that could cause model instability if included in the same model. We identified correlated covariates despite the known robustness of two-sample HSMs to covariate collinearity [49, 50]. We considered these sets of correlated covariates during all phases of model selection, except the first.

We performed HSM model selection in 4 phases. Phase 1 screened covariates in each of our six covariate themes by fitting all theme covariates to the “used” and “available” points at once in Maxent. Strongly correlated covariates in the same group were allowed in the Phase 1 model because Phase 1 simply screened variables based on predictive strength. We assessed the predictive strength of individual covariates using a combination of measures reported by Maxent. We evaluated marginal plots, percent contribution, permutation importance, regularized training gain, and testing gain to assess predictive strength [51]. High strength covariates exhibited a biologically reasonable marginal response, fitting the known and accepted knowledge base, and possessed high relative influence (typically values ≥ 20), as measured by percent contribution and permutation importance. Maxent’s jackknife test measured the goodness of fit of the model. We advanced covariates with jackknife values of approximately 2.5 or better during both regularized training and testing gain. A jackknife value of 2.5 translates to a likelihood improvement of 12.2 (= exp (2.5)) times relative to the null model [52].

During phase 2, we combined the top Phase 1 covariates across groups into a single model but prohibited correlated covariates from appearing in the same model. We defined covariates to be correlated when their pairwise Pearson and Cramer V statistics exceeded 0.65. Due to these correlation prohibitions, we ultimately obtained two HSM models each containing nine pairwise uncorrelated covariates. At the end of Phase 2, we advanced the covariates from each model that exhibited high predictive strength based on percent contribution, permutation importance, regularized training gain, and testing gain.

During Phase 3, we divided the five unique covariates that advanced from Phase 2 into four groups of three uncorrelated covariate groups (Pearson’s R^2^ less than 0.65). We fitted each of the four models and assessed relative quality using the Akaike information criterion (AIC) [53] over 10 bootstrap resamples of the data. We inspected boxplots of AIC over bootstrap iterations and advanced to Phase 4 the model with the lowest median AIC.

During phase 4, we optimized Maxent’s regularization multiplier using the best Phase 3 model. Prior phases had Maxent’s regularization multiplier set at a value of 2. Phase 4 was designed to investigate the predictive strength of multipliers around 2. We tried nine regularization multipliers from 1.0 to 5.0 in steps of 0.5 and chose the value with lowest median AIC over 10 bootstrap resamples.

During all model selection phases, we set Maxent to perform 10 bootstrap replicates each with 5000 fitting iterations. We used scaled logistic output and model form combinations of Linear, Quadratic, Product, and Hinge shapes. During each bootstrap replicate, Maxent fitted the HSM model to a random subset of 80 percent of the selected “used” locations and computed model fit statistics on the remaining 20 percent.

### Habitat classification

**Fig 3:**
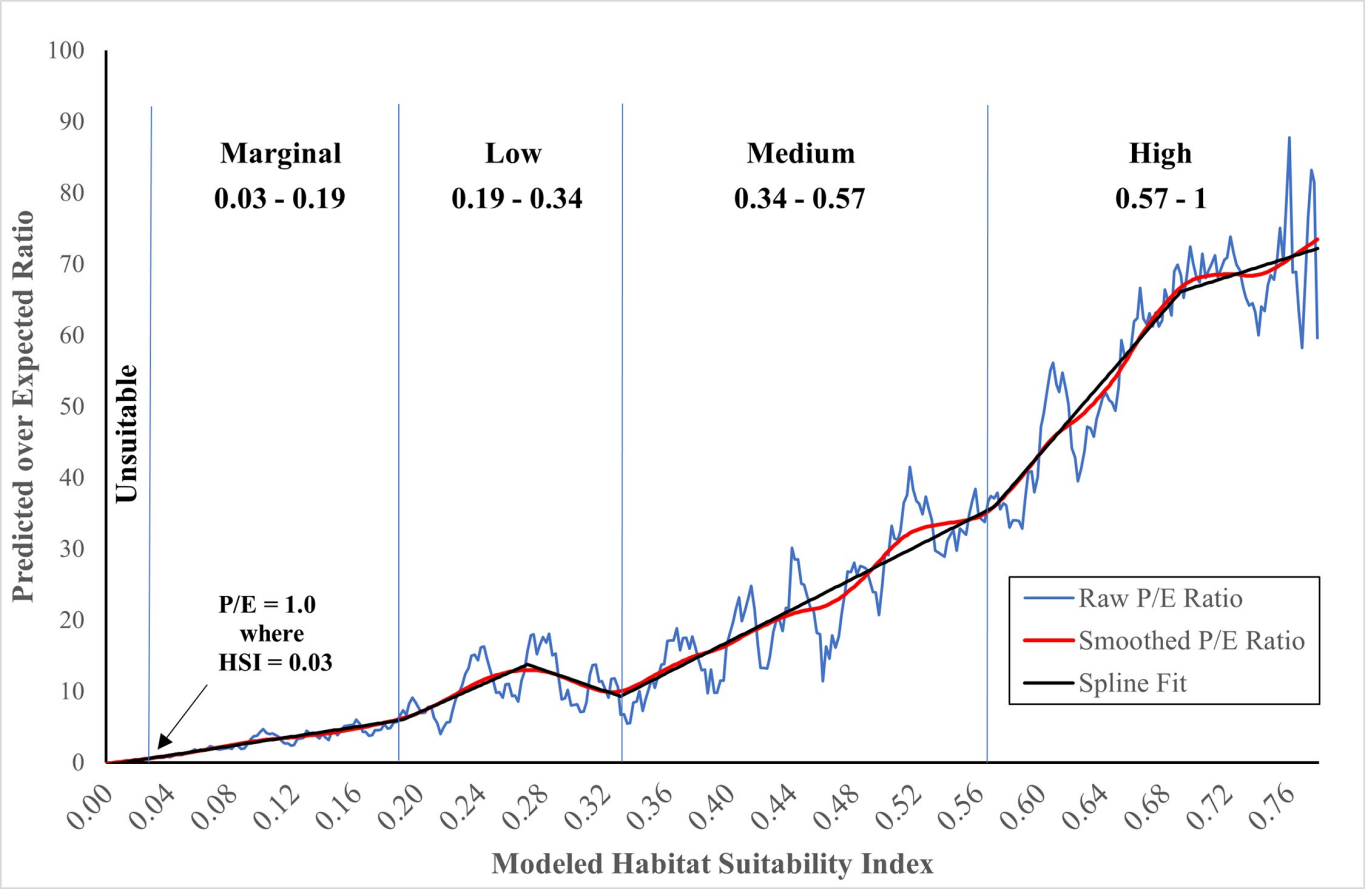
Predicted-to-Expected (P/E) ratio curve. P/E curve from the final HSM model showing the continuous Boyce (raw P/E ratio) curve, smoothed curve [54]; function supsmu in R) and fitted lines from a spline model with kinks at habitat classification breaks.

To classify habitat across the study area, we estimated break points in a smoothed continuous P/E ratio curve [25, 26]. P/E curves provide insight into an organism’s use of the available habitat as predicted by model HSI values. The expectation from a sound result is that as model predicted habitat quality increases use will increase as well. We constructed the P/E curve from our final HSM predictions using overlapping bins (discrete ranges) of predicted HSI values with widths equal to 0.02 centered on values from 0.02 to the maximum prediction, in steps of 0.002. Segments of the P/E curve with different slopes indicate changes in the accumulation of “used” points relative to “available” and represent a change in selection habits relative to the other segments (Fig 3). To identify specific breakpoints, we fitted a piece-wise linear curve via ordinary least-squares to the smoothed P/E curve and searched for the best fitting habitat breakpoints via direct search across all possible breakpoint combinations. We computed breakpoints on a smoothed version of the raw P/E ratios that we smoothed using function “supsmu” in R [54] to aid visual interpretation and speed the search for breakpoints; but the identified breakpoints varied little when we segmented the un-smoothed P/E curve.

We forced the first habitat breakpoint at the maximum HSI value with P/E < 1.0 because those areas contained fewer “used” points than expected by chance [26]. We regarded locations with P/E < 1.0 to be “unsuitable”. For P/E values > 1.0, we combined areas with predicted values between each of the best-fitting breakpoints and attached a descriptive label to the class. Ultimately, we identified four habitat classes for areas with P/E values > 1.0 that we labeled “marginal”, “low”, “medium”, and “high” (see Results). We used these categories to delineate murrelet habitat patches.

### Patch delineation

**Fig 4:**
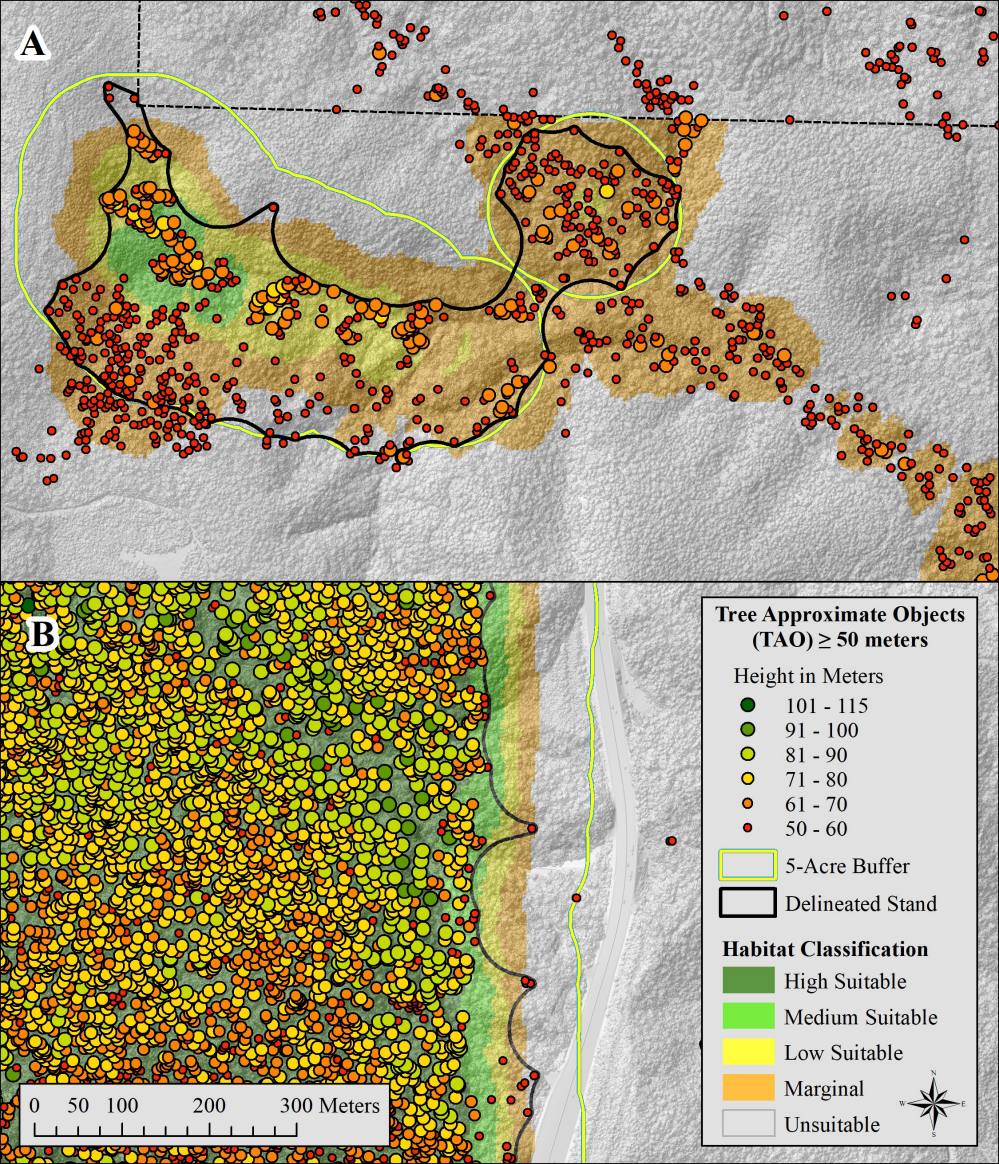
Habitat delineation and TAOs. Map panels show examples of delineated marbled murrelet habitat patches. Patch boundaries (black) were derived by constructing a five-acre (2-hectare, 80.25 meter) buffer around Low or better suitability pixels (HSI values ≥ 0.19, Fig 3), selecting all tree approximate objects (TAO) ≥ 50 meters in height inside the buffer, and computing a concave hull around these trees. TAOs ≥ 50 meters within 80.25 meters of a pixel contributed to predictions of Low and better habitat areas. Panel (A) illustrates a higher quality patch delineated on Green Diamond property within the Plan Area and panel (B) illustrates an area of old-growth forest in Redwood National and State Park between the Plan Area boundary and the ½ mile outer buffer delineating the boundary of the Study Area.

We identified marbled murrelet habitat patches by first mapping habitat classes onto 5-meter raster cells (i.e., pixels) covering the entire study area. We then buffered patches of “low” or better habitat outward by 80.25 meters (263 feet) to encompass areas adjacent to the patch that could have contributed to habitat quality inside the “low” and better patch (Fig 4). Our buffer distance (i.e., 80.25 meters) was the radius of the 2-hectare (5-acre) circular moving windows that were a part of the best model’s TAO-based covariates and hence TAOs within this distance contributed to predictions inside the patch. Within this buffered region, we identified all TAOs ≥ 50 meters. We then placed a concave hull [55] around all TAOs ≥ 50 meters and smoothed its perimeter to construct boundaries around patches of habitat with better-than-random selection characteristics. We computed concave hulls using the concaveman R package [56] with concavity parameter set to 0.8 and default length threshold set to 0.0. We smoothed the perimeter of our concave hulls by computing a 60-meter outward buffer followed by a 55-meter inward buffer. This outward and inward buffering process removed many internal intrusions (bays between peninsulas) when they occurred and thereby made the perimeter both more conservative (larger), realistic, and easier to follow in the field.

### Model evaluation

We used the receiver operating characteristic curve (ROC) and area under curve (AUC) output from Maxent to evaluate overall model prediction ability. AUC ranges from 0 to 1, with a value of 0.5 indicating predictions no different than random chance and with AUC = 1 indicating perfect discrimination. In addition to AUC, we computed the sensitivity of our model to correctly predict “used” locations given a specific threshold above which locations were considered “habitat”, and below which locations were considered “not habitat”. Using that same threshold, we computed the Positive Predictive Value [57] and Cohen’s Kappa [58, 59] to assess the predictive value of our HSM. We evaluated marginal plots of relative probability of selection (i.e., HSI value) by each covariate in the final model to gain insight into model predictions and to assess the biological reasonableness of predictions in each dimension.

## Results

### HSM model selection

The six Maxent runs completed during Phase 1 (covariate screening) yielded eleven covariates (S2 Table in S1 File). We advanced three covariates from the TAO Point Density and TAO Point Statistics themed groups, two from the Biogeographic/Geographic group, and one each from the TAO Percentiles, Canopy metrics, and Climate groups. At the end of Phase 1, we discounted two covariates (Coast_Dist and PPT) with high influence because they exhibited implausible bimodal marginal plots at the extremes of their ranges. We ultimately discounted another variable (ZCI) that was initially elevated to Phase 2 but exhibited very low Permutation Importance and regularized training/testing gain. The categorical nature of the ZCI variable, essentially defining the coastally influenced fog zone, provided little spatial discrimination in our study area.

After separating correlated covariates, we fitted two Maxent models during Phase 2 of model selection (S2 Table in S1 File). Both models contained nine uncorrelated variables. We identified top variables in both models, removed duplicates among the top models, and advanced five covariates from Phase 2 to 3. All five advancing covariates came from the TAO-based density and height themed groups. Covariates in the percentile and canopy metric groups, as well as those in the biogeographic, and climate groups displayed little predictive value compared to the TAO-based density and height covariates. In this stage, the Simpson Diversity Index for TAO height classes (SDI_3cl_5ac) appeared to perform well exhibiting a high Percent Contribution but yielded a very low Permutation Importance. During ancillary Phase 2 investigations, we discovered computational inconsistencies in the Simpson Diversity Index results and dropped this covariate.

**Fig 5:**
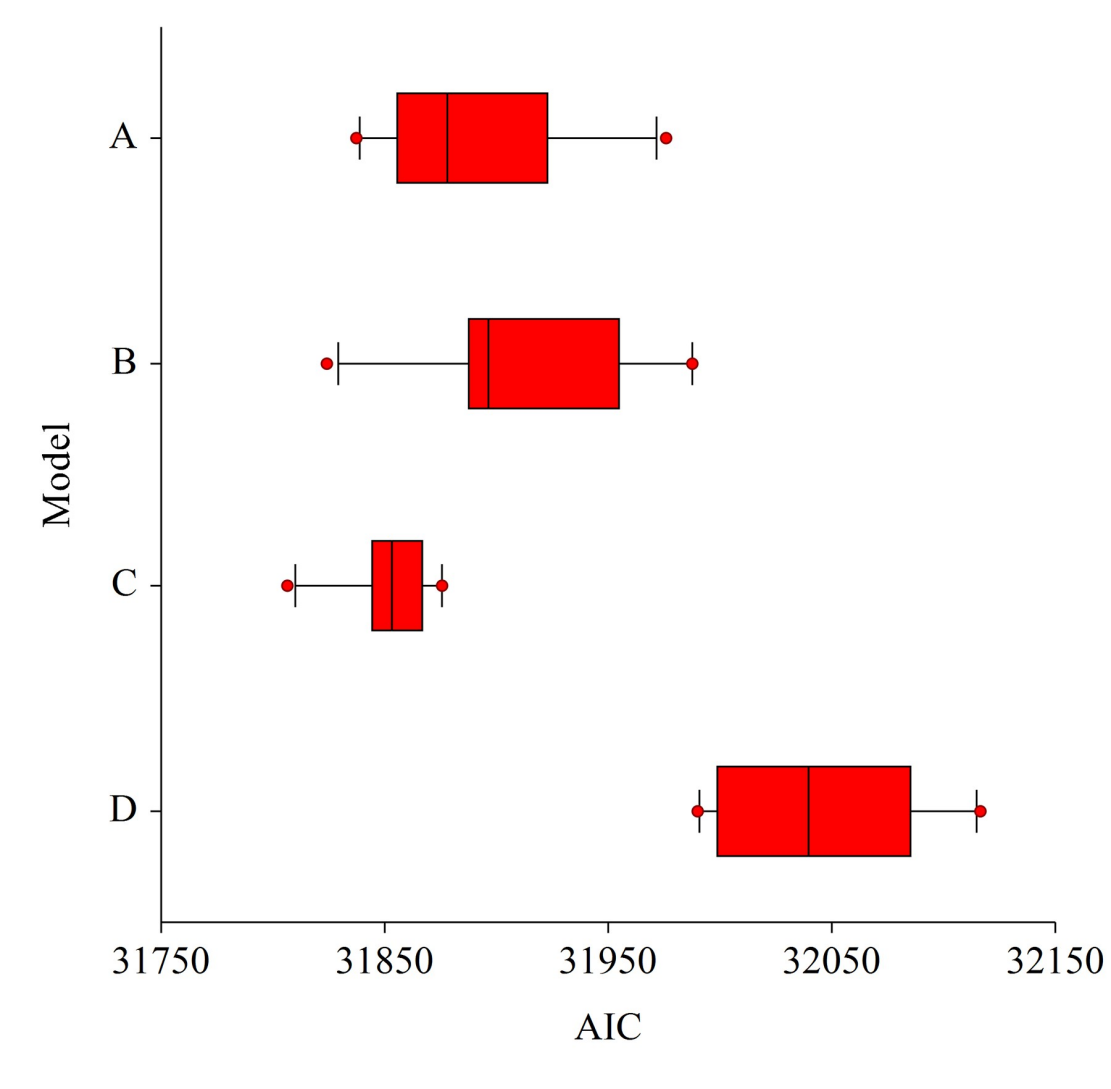
AIC plot - Final model selection. Box and whisker plots of Akaike Information Criteria (AIC) values for the four models with three-variable combinations. The best three-variable model (C) consisted of the point density of tree approximate objects (TAO) ≥ 60m within a 5ac buffer, the sum of heights of TAO ≥ 50m within a 5ac buffer, and the standard deviation of TAO ≥ 50m within a 5ac buffer. Median and dispersion for model C are the lowest suggesting both parsimony and consistency between replicates. Box boundaries are at the 25th and 75th percentiles, the whiskers extend from the 10th to the 25th and the 75th to the 90th percentiles, and the outliers (red dots) are represented beyond the whiskers.

During Phase 3, we fitted four models that represented all possible models of uncorrelated covariates (S2 Table in S1 File). Among these four, the model containing density of TAOs ≥ 60 meters, total height of TAOs ≥ 50 meters, and the standard deviation of TAO heights ≥ 50 meters produced the lowest median AIC (ΔAIC to next best model = 39.3). This model also produced the least variability in AIC among the four models tested (Fig 5), suggesting stability and consistency among random replicates. Among covariates in this model, tree density (TAOs ≥ 60 meters) and total height (of TAOs ≥ 50 meters) contributed more to predictions than the standard deviation of tree height (of TAOs ≥ 50 meters).

**Fig 6:**
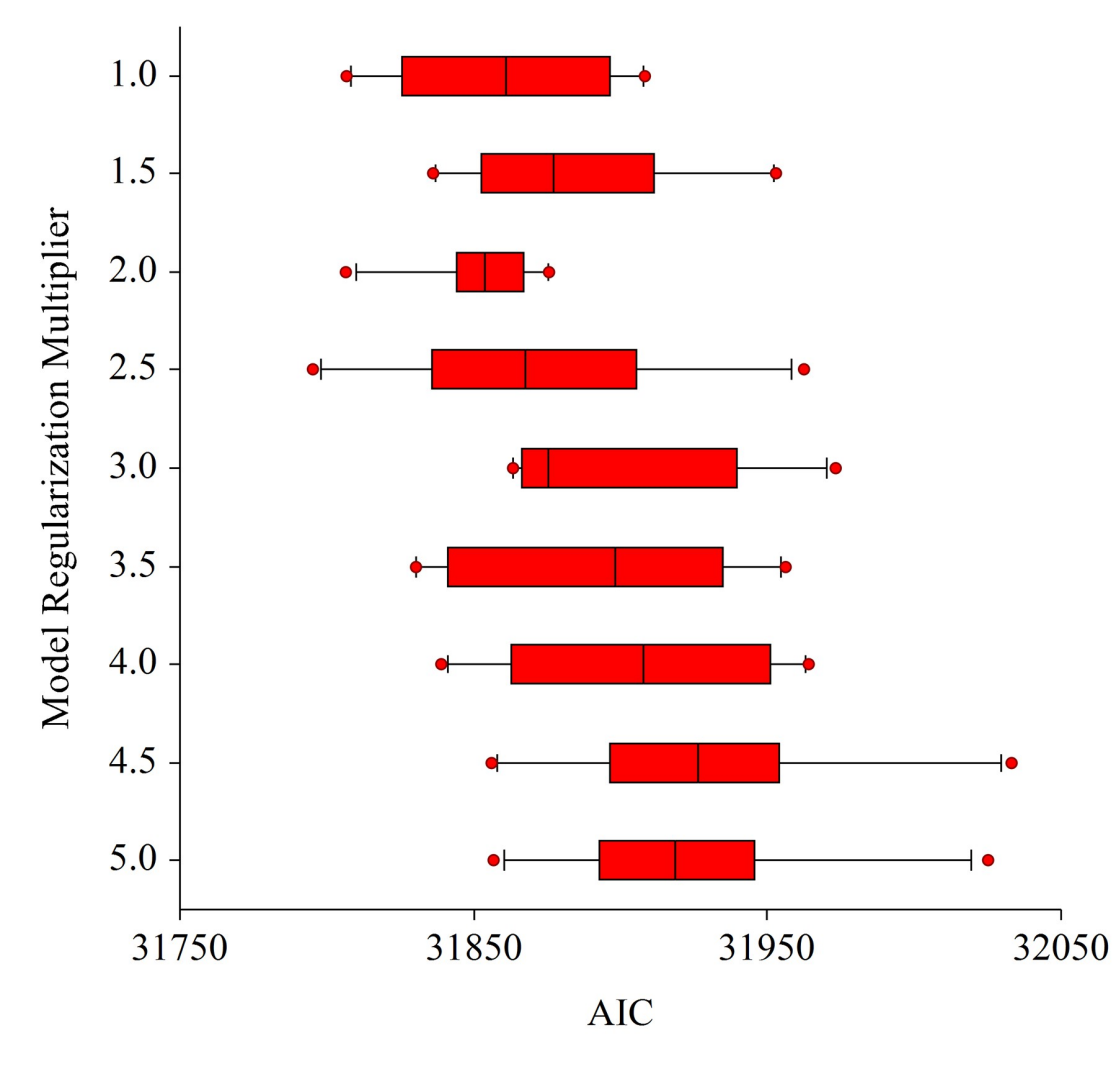
AIC plot - Regularization multiplier assessment. Box and whisker plots of Akaike Information Criteria (AIC) values for the best three-variable model (C) with regularization multiplier varied from 1.0 to 5.0 in 0.5 increments. Median and dispersion for the model with RM = 2.0 are the lowest suggesting both parsimony and consistency between replicates. Box boundaries are at the 25th and 75th percentiles, the whiskers extend from the 10th to the 25th and the 75th to the 90th percentiles, and the outliers (red dots) are represented beyond the whiskers.

We ran Maxent nine times during Phase 4 to identify the best regularization multiplier (RM) between 1.0 and 5.0. The Maxent run with RM equal to 2.0 produced the lowest AIC among those tested and the least amount of variation over bootstrap replicates (Table 1, Fig 6). We set RM to 2.0 during covariate selection (Phases 1 through 3) and we were not surprised that RM = 2.0 produced the lowest AIC because we found in prior Maxent modeling efforts that an RM = 2.0 is a good compromise between model complexity (lower RM values) and simplicity (higher RM values). We acknowledge that using a different RM during Phases 1 through 3 could conceivably have resulted in a different best-fitting model. Regardless, the minimum AIC with RM = 2.0 supports the idea that no other combination of RM and covariates in our final model performed better, and subsequent model evaluation indicated that our final model adequately fitted the “used” and “available” data.

**Table 1:** AIC based regularization multiplier assessment. The results of nine iterative replicated model runs of the best candidate (3var_C) resulting from varying the Regularization Multiplier (RM) in Maxent from 1 to 5 by steps of 0.5 and listed by Akaike Information Criteria (AIC) values. The RM value of 2.0 exhibited both the lowest AIC value and least AIC dispersion between replicates followed by a non-competitive RM of 1.0 (ΔAIC of 6.8).

Regularization Multiplier	Average Number of Model Parameters	Average of log Likelihood	Average AIC	Delta AIC
2.0	36.7	-15889.3	31852.0	
1.0	42.7	-15886.7	31858.7	6.8
2.5	34.7	-15900.5	31870.5	18.5
1.5	38.7	-15902.9	31883.3	31.3
3.5	30.0	-15916.7	31893.4	41.4
3.0	33.5	-15913.8	31894.7	42.7
4.0	34.3	-15918.1	31904.7	52.7
5.0	30.2	-15932.0	31924.5	72.5
4.5	33.6	-15931.6	31930.4	78.5

**Fig 7:**
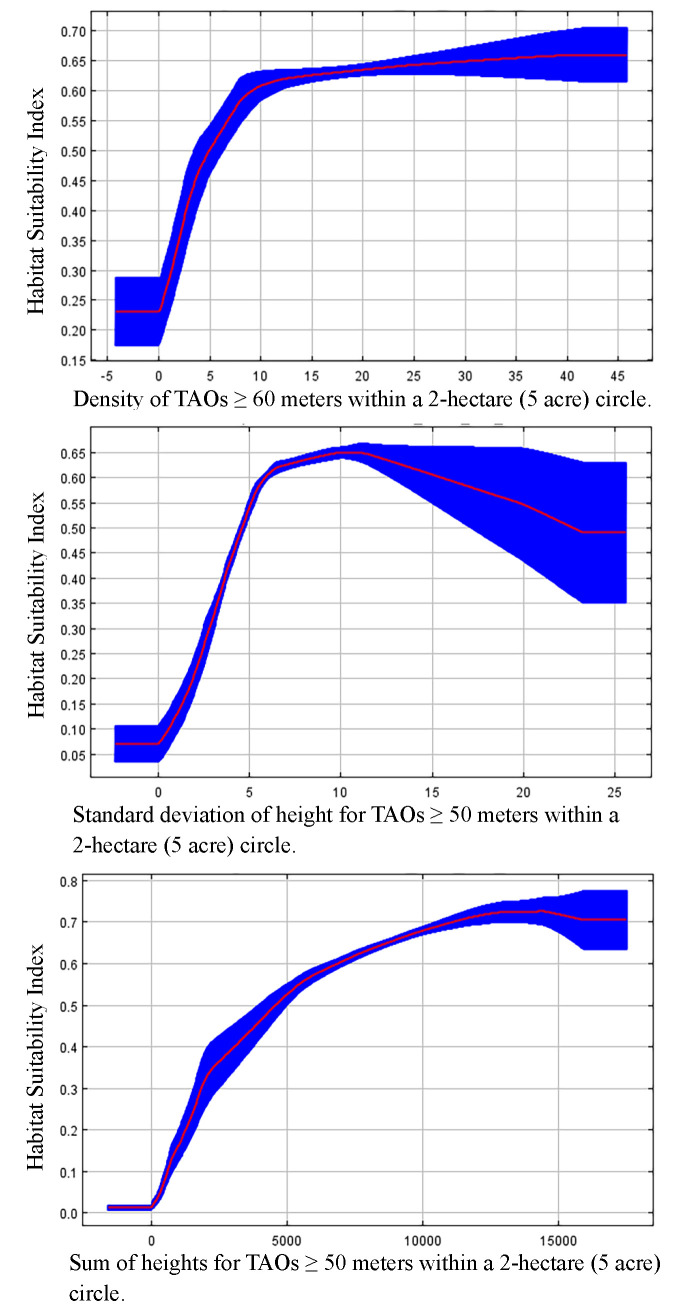
(Top, Middle, and Bottom) Final model covariate response plots. Marginal plots showing response of relative habitat suitability to final model covariates: (Top) Density per acre of TAOs ≥ 60 meters tall within a 5-acre circle, (Middle) Standard deviation in height for TAOs ≥ 50 meters tall within a 5-acre circle, and (Bottom) Sum of heights for TAOs ≥ 50 meters tall within a 5-acre circle. The red line represents the mean value for 10 replicate runs and the blue shade is ± one standard deviation.

Marginal plots for the three TAO-based density and height covariates in our final model (Fig 7) revealed positive correlations between relative probability of selection and all three measures. HSI values associated with TAO density increased linearly from 0 (no trees ≥ 60 meters) to 25 tall trees per hectare (10 trees per acre) in a surrounding 2-hectare (5-acre) circle. HSI values associated with standard deviation of TAO height increased linearly from 0 to 6 meters (19.7 feet) in a 2-hectare (5-acre) circle, respectively. Beyond those levels, relative probability of selection reached a maximum at 10 meters, then decreased. For the sum of individual TAO heights within the surrounding 2-hectare (5-acre) area, HSI values increased curvilinearly, plateauing around a sum of 13,000 meters, without appreciable change to 15,000 meters.

### Habitat classification

The continuous P/E curve for our study showed a generally increasing trend from 0.0 up to the maximum HSI value of 0.78 (Fig 3). Our P/E ratio exceeded 1.0 for HSI values greater than 0.03; hence we considered areas with HSI <= 0.3 as “unsuitable” The best fitting break point model for HSI values above 0.03 included five break points at 0.19, 0.27, 0.34, 0.57, and 0.69. The P/E segment between 0.27 and 0.34 identified P/E ratios with negative slope in HSI value. Decreasing P/E on this segment may be due to removal of “used” locations within 20 meters of “used” patch edges that we implemented to reduce edge effects. We consider the brief and small decrease in P/E in this segment an anomaly in an otherwise monotonically increasing curve and immaterial to management implications; consequently, we removed the 0.27 breakpoint thereby combining the adjacent categories. In addition, we discarded the breakpoint at 0.69 because it fell at the highest end of the suitability curve where the slope of the line flattens and hence imparted little management concern. The final set of breakpoints delineated the five habitat classes which we labeled unsuitable (0 – 0.03), marginal (0.03 – 0.19), low (0.19 – 0.34), medium (0.34 – 0.57), and high (0.57 – 0.78). We note that a large proportion of the “marginal” areas were adjacent to and dependent upon areas of “low” or better habitat. That is, without the supporting TAOs in nearby “low” or better habitat, a large proportion of our “marginal” habitat would have been deemed “unsuitable” (Fig 4).

**Fig 8:**
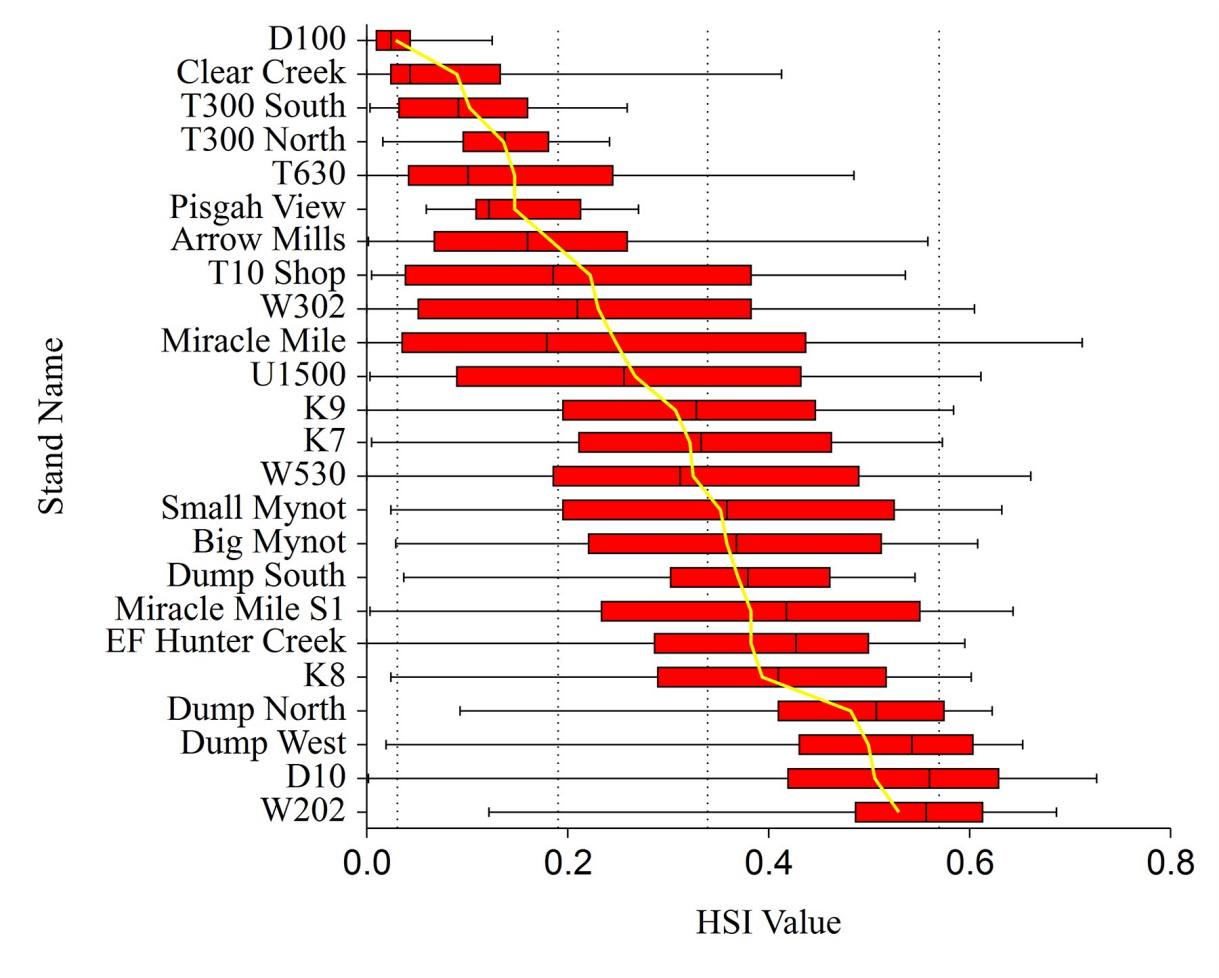
Mean and median HSI values for historic murrelet stands. Box and whisker plots showing mean and median Habitat Suitability Model (HSM) values of 24 historic forest stands containing observed marbled murrelet nesting behavior. Box boundaries are at the 25th and 75th HSM percentiles, the whiskers extend to the extents of the range, and the yellow line represents the mean HSM value. Vertical dashed lines represent the boundaries of the derived habitat classification; unsuitable (0 - 0.03), marginal (0.03 - 0.19), low (0.19 - 0.34), medium (0.34 - 0.57), and high (≥ 0.57).

Under our classification scheme, the study area, and smaller Plan Area were heavily dominated by unsuitable habitat (96.01% and 98.08% respectively) due to the scarcity of TAOs ≥ 50 meters (Table 2). The remainder of the Study and Plan Areas are mostly marginal (2.36% and 1.63%), with less than 0.7% in the low, medium, or high classifications. We found that 20 of the 24 historic known used patches in the Plan Area contained at least one HSI value in the medium or high habitat suitability index range, while 17 of the 24 contained average HSI values in the low or better classes (Fig 8).

**Table 2:** Summary of habitat classes by plan and study area. Areal extant and percentage within each marbled murrelet habitat class (HSM raster), along with relative habitat suitability index (HSI) range, on the Plan and Study area.

		Plan Area		Study Area	
Habitat Class	HSI Range	Hectares (Acres)	Percent	Hectares (Acres)	Percent
Unsuitable	0.0 – 0.03	142,319 (351,678)	98.08%	228,847 (565,491)	96.01%
Marginal	0.03 – 0.19	2,370 (5,856)	1.63%	5,617 (13,880))	2.36%
Low	0.19 – 0.34	243 (601)	0.17%	1,106 (2,732)	0.46%
Medium	0.34 – 0.57	143 (354)	0.10%	1,155 (2,855)	0.48%
High	0.57 – 0.78	24 (60)	0.02%	1,630 (4,028)	0.68%
Total		145,100 (358,549)	100.00%	238,355 (588,987)	100%

### Patch delineation

**Fig 9:**
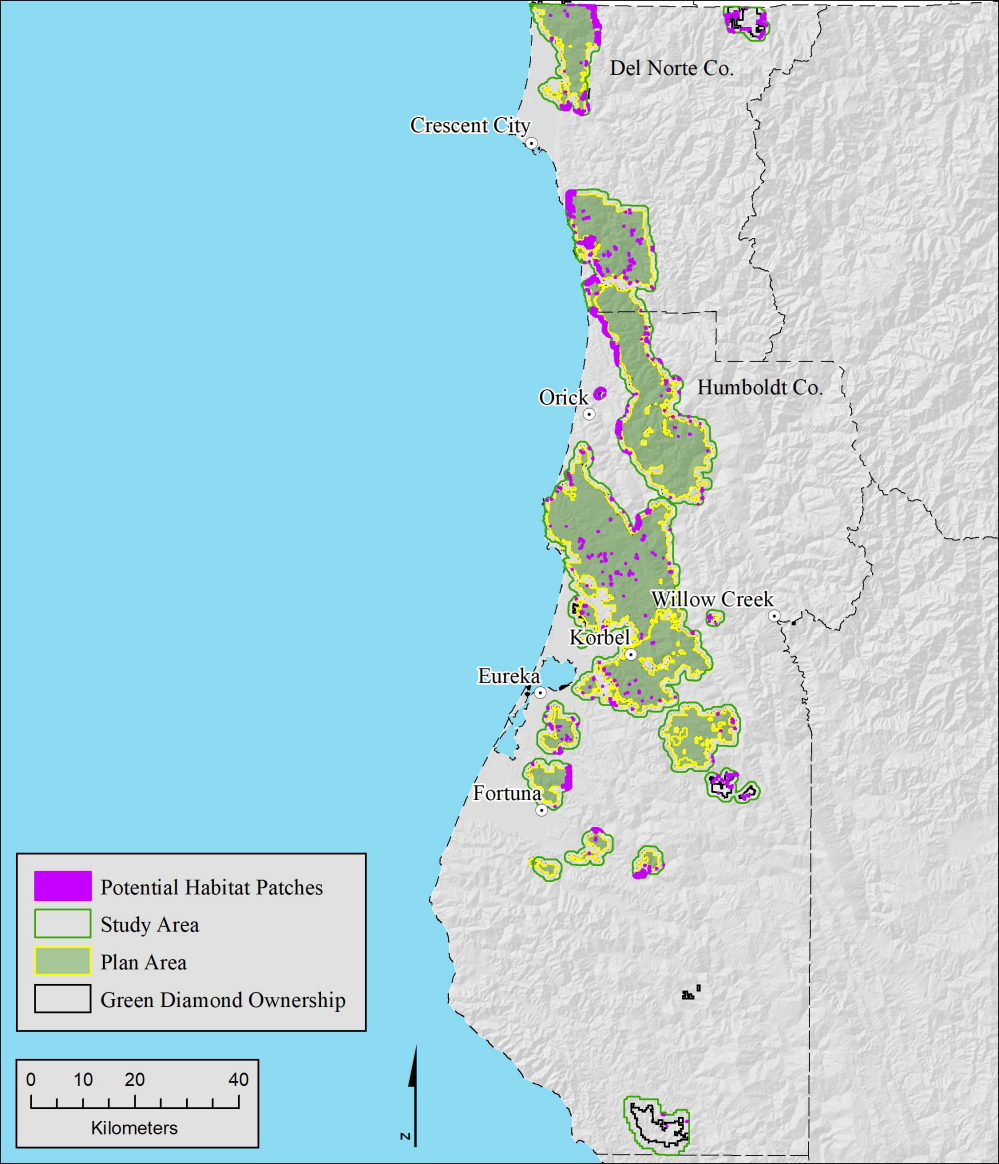
Potential habitat across the study area. Distribution of potential habitat patches within the Study Area. Patches were identified via a concave hull process applied to all TAO’s within a five-acre buffer (80.25m radius) of areas identified as low suitable or better (HSI ≥ 0.19) by the Maxent model.

In the Study Area, we identified 312 patches encompassing 5,421 hectares (13,395 acres; Fig 9). Similarly, within the smaller Plan Area, we identified 171 patches, encompassing 729 hectares (1,802 acres), that received classifications of low suitability or better (Table 3). Within the Plan Area, twenty-four of the 171 patches occurred in or adjacent to known “used” patches. An additional 34 patches in the Plan Area were near boundaries where most of the contributing TAOs were located just outside of the Plan Area but still in the Study Area (e.g., Prairie Creek Redwoods State Park).

**Table 3:** Summary of model delineated habitat. The values represent the model predicted area by suitability classes and occupancy status, rounded to the nearest whole hectare, based on the raw HSM raster. “Used” patches are those with documented occupied behaviors, and within the Study Area include patches on Federal and State parks, Bureau of Land Management reserves, unharvested old growth private managed forests, partially harvested old growth forests, and younger (< 70 years old) second and third growth forests with residual old growth trees. “Available” patches are areas with non-confirmed Occupancy (Occupied behaviors) status. Both “Used” and “Available” are delineated by methods described in the text.

** **	**Plan Area (Green Diamond only)**					
** **	**Unsuitable**	**Marginal**	**Low**	**Medium**	**High**	**Plan Total**
Used	16	86	70	96	24	292
Available	21	270	118	29	0	437
Total	37	356	188	125	24	729
	**Study Area (Green Diamond plus ½-mile off-property buffer)**					
	**Unsuitable**	**Marginal**	**Low**	**Medium**	**High**	**Study Total**
Used	109	377	339	627	1,465	2917
Available	172	1,083	620	466	163	2504
Total	281	1,460	960	1092	1628	5,421

In the Plan and Study Areas, polygon patches we considered occupied comprised 89.9% and 99.5%, respectively, of the raster-based high habitat classification, 54.2% and 66.8% of the medium classification, and 30.7% and 28.9% of the low classification. Not surprisingly, these figures indicate that most of the better murrelet habitat occurred in the patches we considered occupied, and hence few high-quality patches exist outside our already identified “used” patches. Small areas of low, medium, or high suitability were occasionally left outside of concave hull delineated patches because they lacked 50-meter and taller trees (Fig 4).

### Model evaluation

**Fig 10:**
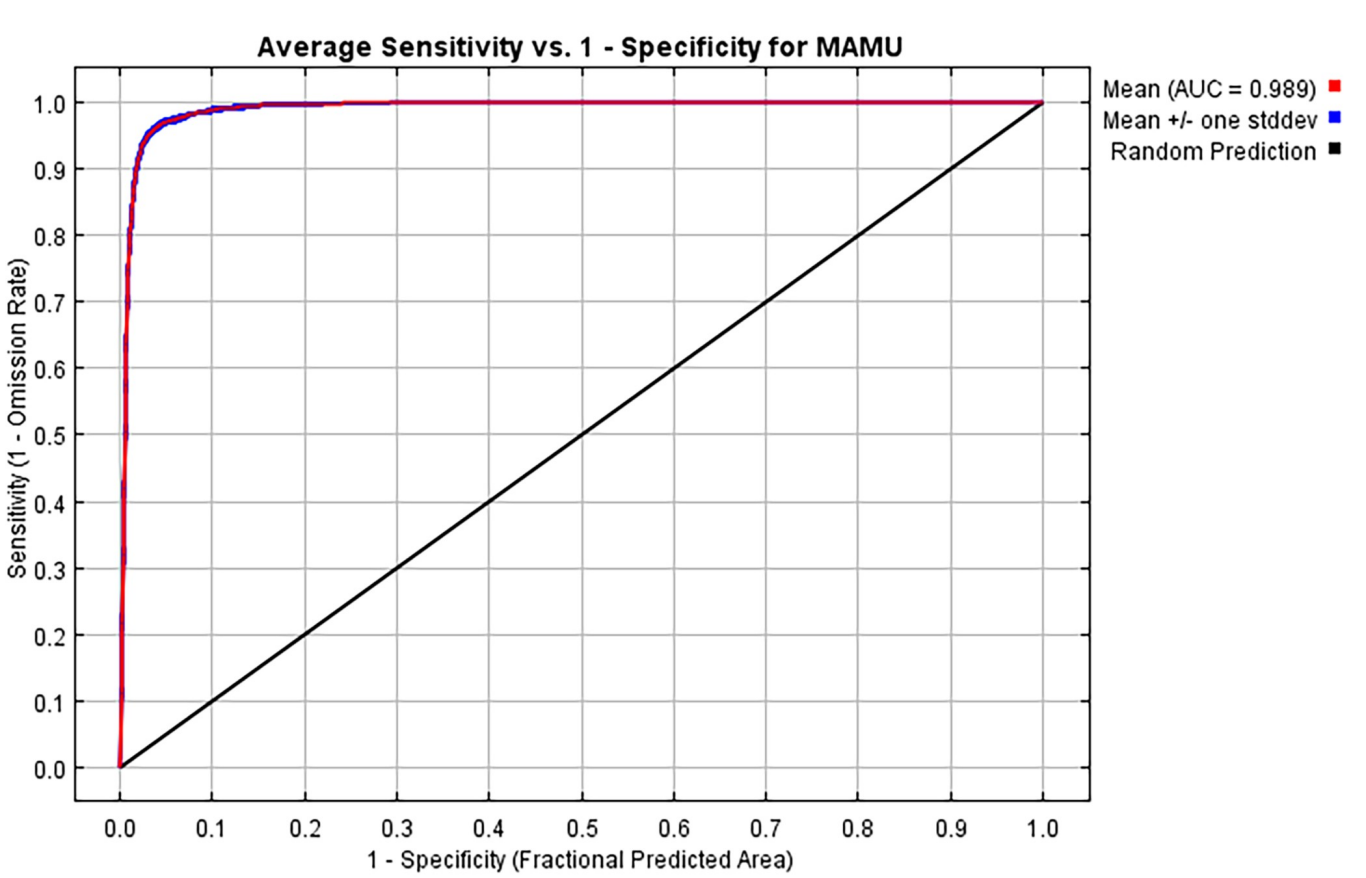
Receiver Operating Characteristic (ROC) curve. This ROC curve is an average of 10 replicate runs. The average training Area Under the ROC Curve (AUC) for replicate runs is 0.989, and the average standard deviation is 0.001. The best model in our analysis predicting the environmental niche of marbled murrelets for the study area had an AUC value of 0.989 and exhibited very low deviation from the mean of the ten replicates over the full ROC curve. This value indicates a model with high predictive ability and low probability of false negatives.

Our final HSM model attained an AUC value of 0.99 (Table 4, Fig 10). Furthermore, AUC varied little over the ten bootstrap replicates evaluated by Maxent. AUC = 0.99 indicates a model with very high predictive ability and low probability of false negatives. We adopted the lower limit of the Low habitat class (i.e., HSI = 0.19) as the threshold above which we classified an area as murrelet habitat. With this threshold, our model achieved a sensitivity value of 0.89 indicating that nearly all “used” locations had HSI values above the threshold and were predicted to be habitat by the model. Our model attained a Positive Predictive Value (PPV) of 0.49, indicating a significant number of “available” locations (987) with HSI values above the threshold (0.19). Our model achieved a Cohen’s Kappa value of 0.63 and indicated “substantial” agreement between the predicted habitat areas and known “used” locations if we adopt the guidelines of Landis and Koch [60]. Other authors consider Cohen’s Kappa = 0.63 to be “good” agreement [61, 62].Table 5 presents these results in a confusion matrix format, reinforcing the metrics from Table 4.

**Table 4:** Model performance measures. Predictive performance measures for the final model of marbled murrelet habitat using training data and HSI threshold of 0.19. Sensitivity is the number of correctly predicted used locations divided by the total number of locations predicted to be “habitat” (i.e., TP / (TP + FN) = 956 / 1,080). Positive predictive value is number of correctly predicted used locations divided by total number of used locations (i.e., TP / (TP + FP) = 956 / (956 + 987). Cohen’s Kappa measures the agreement between known and predicted values and is defined as 2(TP * TN – FN * FP) / (TP + FP) * (FP + TN) + (TP + FN) * (FN + TN)) (i.e., 221,044,368 / 351,924,612).

Performance Metric	Metric Value
Area under the curve (AUC)	0.99
Sensitivity - True Positive Rate (TPR)	0.89
Positive Predictive Value (PPV)	0.49
Cohen’s Kappa (k)	0.63

**Table 5:** Confusion matrix. Contingency table showing the true positives, false positives, false negatives, and true negatives based on the “used” and “available” model input and the predicted model output.

	True Positive (Used)	True Negative (Available)	Total
Predicted Positive (Used)	TP = 956	FP = 987	1,943
Predicted Negative (Available)	FN = 124	TN = 115,737	115,861
Total	1,080	116,724	117,804

## Discussion

Our “used” habitat records on private timberland were derived from focused surveys conducted by trained individuals after input and guidance from regulatory agencies. We surveyed a wide and representative range of environmental conditions within the study area to establish used patches because these surveys were necessary to obtain timber harvest permits. Our BAS sample minimized sampling bias of background values because points came from a spatially balanced sample of environmental conditions within and outside of known occupied habitats.

Within the California range of marbled murrelets, only 26 nest trees have been reported and only 1 fell on private timberland in our study area. These 26 nest trees were used in analyses conducted for the Northwest Forest Plan [28], and these authors supplemented their small sample size by including additional information from eggshell fragments (n=56), downed young (n=11), and an equivalent total number of occupied sites (n=93). Like Hamer and Nelson [14] and Lorenz et al. [28], we used several sources of information to determine habitat use. We sourced most information from inland audio-visual surveys conducted on private timberland prior to timber harvest plan approvals from 1992 to 2021. During this period, we conducted audio-visual surveys in all areas that the landowner, state, and federal agencies agreed could potentially be murrelet habitat. Hence, the surveyed areas covered all potential murrelet habitat based on the prevailing knowledge of habitat during the study period. In total, we surveyed 26 such areas and our model ended up classifying these areas as ranging from unsuitable to medium, with most classified as unsuitable or marginal.

We do not know the degree to which detection probability varies with the covariates influencing the relative probability of occupancy [63]. However, on private timberlands we used data from surveys designed to clear areas for timber harvest and that were designed to detect murrelet presence with 95% or greater accuracy. Because of this, we discount the possibility that a large number of murrelets were not detected on private timberlands and that the rare non-detection depended on covariates affecting selection. We also discount the possibility that high densities of large trees reduce detection probability because the audio-visual surveys were specifically designed to detect murrelets in these situations.

We used a very large “available” sample which has been noted to artificially improve AUC simply because it is partially dependent on the ratio of “used” to “available” points [63]. Our large “available” sample was necessary to adequately quantify the distribution of extremely patchy and sometimes rare environmental conditions relevant to marbled murrelets. To assess whether our large “available” sample artificially inflated AUC, we refitted the final model after randomly reducing the “available” sample from 117,804 to 11,828 records (10.04% of the original). The refitted final model yielded an AUC of 0.987 compared to the original model’s AUC = 0.989. Likewise, we simulated the effects of smaller “available” samples on Cohen’s Kappa by successively halving the “available” sample. Contrary to expectations, Kappa increased from 0.63 (117,804 “available” points) to a maximum of 0.90 (7,295 “available” points – close to the Maxent default of 10,000) then decreased to 0.72 (228 “available” points). While variable, all simulated Kappa values suggest substantially better prediction than random classification. Generally, Kappa (κ) values less than 0.4 are considered poor, 0.4 < κ < 0.75 are considered good, and κ greater than or equal to 0.75 excellent [58, 60–62]. Our reported value of 0.63 falls in the “good” range.

**Fig 11:**
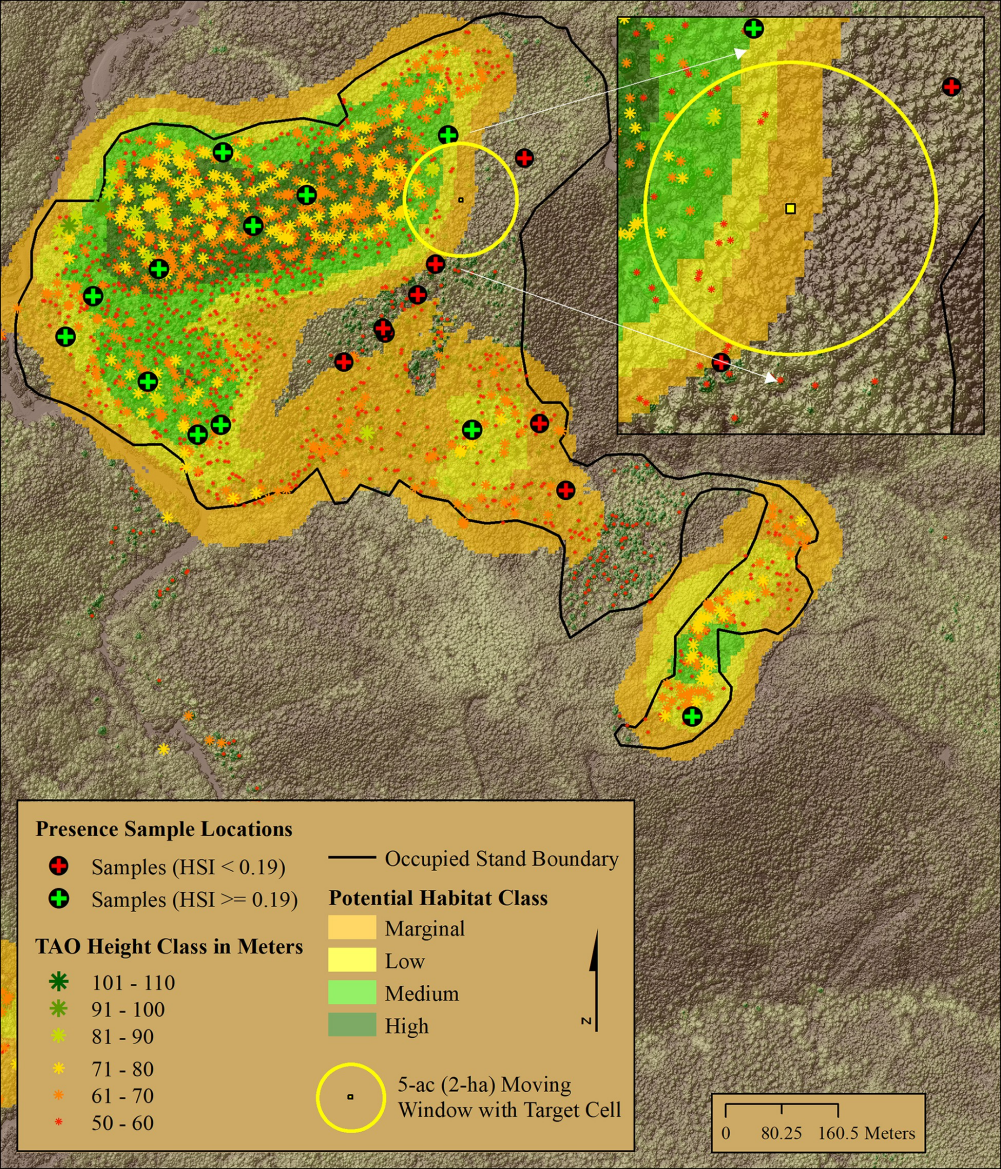
BAS training samples. An example of BAS sample locations used to train the Maxent model, TAOs ≥ 50 meters tall, potential habitat classifications, and 5-acre circular moving window with central target cell. As the 5-acre circular window moves from a core area containing higher densities of TAOs ≥ 50 meters to non-habitat area with low densities or no TAOs ≥ 50 meters outside the occupied stand, the modeled HSI values calculated for the target cell decrease as a result. This heterogenous distribution of TAOs ≥ 50 within occupied stands results in internal pockets and areas adjacent to the occupied stand edges with HSI values below the threshold value of 0.19 leading to perceived errors of omission (black circle/red +).

Our HSM scored very high to reasonably well on the predictive accuracy measures we computed. The principal issue impeding higher sensitivity scores is the existence of used locations with HSI values below the threshold of 0.19 (i.e., false negatives) affecting approximately 11% of our “used” sample. Two conditions resulted in false negatives. The foremost was due to forest heterogeneity within the occupied patches falling mostly on managed timberlands. Within the “used” patches, internal and edge inclusions of low-quality habitat (sparse large trees) with HSI values below the threshold were present in approximately 8% of the used sample. A less important effect occurred near the abrupt edge between old trees and surrounding young forest. Model selection supported a 2-hectare (5-acre, 80.25-meter radius) moving window to calculate predictor variables resulting in gradual decreases in HSI values as the circular window moved out of presumed nesting patches and into adjacent younger forest (Fig 11). This decline near patch edges results in approximately 3% of our used locations near patch edges being associated with HSI values below the threshold.

PPV is directly affected by the presence of predicted habitat outside of the “used” or known occupied patches. Our relatively low PPV value is typical in “used” - “available” HSM predictive efforts where the extent of true occupancy is likely greater than known prior to modeling and when the sample of used locations is small relative to the study area.

Our correlative HSM model indicated that fine-scale, LiDAR-based, individual tree covariates were the best predictors of habitat suitability for marbled murrelets. We summarized the height and density of our derived individual trees (TAOs) at two different scales, 1-acre, and 5-acre, using moving window methods that best represent existing known and potential habitat patches within the study area. Synthesizing individual tree metrics using moving window methods is a compromise between selecting too small an area, and detail is lost, too large an area and resolution is lost. Exploratory assessment of known murrelet stands suggested areas less than 1-acre increased noise due to small sample size (no TAOs within the circle) and areas greater than 5 acres failed to resolve the clear boundaries between previously mapped habitat and the surrounding young managed timberlands. During model selection, the 5-acre scale outperformed covariates compiled at the 1-acre scale and no climatic or biographic/geographic themed variable performed well enough to be included in the final phase. We conclude that the 5-acre scale is a better predictor of marbled murrelet habitat within managed landscapes where environmental covariates are patchily distributed and more dispersed than in large homogeneous old growth stands. We hypothesize that low variation in the climatic, biographic, and geographic variables at a study area scale rendered these covariates ineffective as predictors. We suspect habitat selection could be associated with natural geographic factors such as topographic position and distance to coast (which is positively correlated with elevation) as found in other studies [64, 65]; however, the entire relevant range of geographic and topographic conditions was not well represented in our study and hence the variable selection procedure did not include these factors in the model.

In Oregon, Hagar et al. [64] used a suite of Gradient Nearest Neighbor (GNN) covariates and LiDAR derived covariates to develop a logistic regression model predicting relative probability of marbled murrelet occupancy in similar aged forest stands that were all considered potential habitat based on age (> 80 years) and structural characteristics. They found that the model with five LiDAR derived variables along with distance to the coast performed better than the model with GNN derived variables. The LiDAR variables they used were better predictors of habitat for marbled murrelets at stand and watershed scales when applied to independent data sets, although overall performance was low on the independent data set. They interpreted the LiDAR-derived variables in their model as cover in the upper portion of the canopy, maximum tree height, maximum height of the bottom of the canopy, variation in vertical stratification of the canopy, and distribution of variation across canopy height intervals. These findings are consistent with studies that found multi-layered old forests provide the components necessary to support nesting by marbled murrelets [14, 17, 66]. Clyde [65] assessed the use of individual tree objects, modeled from LiDAR to calculate the local Moran’s I spatial statistic (LMI), to identify the presence of vertically isolated trees and or clusters of tall trees. While the LMI metric yielded low predictive power, canopy height was shown to be important for the highest ranked areas.

Finally, our study advances understanding of marbled murrelet habitat use by incorporating fine scale LiDAR derived individual tree metrics (height, height diversity and density) directly into predictive covariates. All three LiDAR variables included in our HSM are more direct measures of small-scale marbled murrelet habitat requirements than indirect area-based point cloud metrics derived at various raster scales and resolutions. However, because our data contains information on tall trees ≥ 50 meters, field assessments of the presence, quantity, and quality of platforms may facilitate identification of habitat because young tall trees might not provide suitable platforms for nesting [67]. In the future, LiDAR point cloud data may provide platform information on individual trees that will inform future marbled murrelet HSMs.

## Supporting information

S1 File (ZIP)
